# What is the impact of aminoglycoside exposure on soil and plant root-associated microbiota? A systematic review protocol

**DOI:** 10.1186/s13750-022-00274-y

**Published:** 2022-05-12

**Authors:** Jessica Coates, Kathleen J. Bostick, Brooke A. Jones, Nymeer Caston, Mentewab Ayalew

**Affiliations:** 1 Microbiology and Molecular Genetics Graduate Program, Graduate Division of Biological and Biomedical Sciences, Emory University, Atlanta, GA 30329 USA; 2 Department of Biology, Spelman College, 350 Spelman Lane, Atlanta, GA 30314 USA; 3 Division of Natural Sciences and Mathematics, Miles College, 5500 Myron Massy Blvd, Fairfield, AL 35064 USA; 4 Department of Biological and Environmental Sciences, Alabama A&M University, 4900 Meridian Street N, Huntsville, AL 35811 USA; 5 Biology Department, Spelman College, 350 Spelman Lane, Atlanta, GA 30314 USA

**Keywords:** Microbial composition, Resistome, Pathogen suppression, Plant root microbiota

## Abstract

**Background:**

Aminoglycosides are potent bactericidal antibiotics naturally produced by soil microorganisms and are commonly used in agriculture. Exposure to these antibiotics has the potential to cause shifts in the microorganisms that impact plant health. The systematic review described in this protocol will compile and synthesize literature on soil and plant root-associated microbiota, with special attention to aminoglycoside exposure. The systematic review should provide insight into how the soil and plant microbiota are impacted by aminoglycoside exposure with specific attention to the changes in the overall species richness and diversity (microbial composition), changes of the resistome (i.e. the changes in the quantification of resistance genes), and maintenance of plant health through suppression of pathogenic bacteria. Moreover, the proposed contribution will provide comprehensive information about data available to guide future primary research studies. This systematic review protocol is based on the question, “What is the impact of aminoglycoside exposure on the soil and plant root-associated microbiota?”.

**Methods:**

A boolean search of academic databases and specific websites will be used to identify research articles, conference presentations and grey literature meeting the search criteria. All search results will be compiled and duplicates removed before title and abstract screening. Two reviewers will screen all the included titles and abstracts using a set of predefined inclusion criteria. Full-texts of all titles and abstracts meeting the eligibility criteria will be screened independently by two reviewers. Inclusion criteria will describe the eligible soil and plant root-associated microbiome populations of interest and eligible aminoglycosides constituting our exposure. Study validity will be evaluated using the CEE Critical Appraisal Tool Version 0.2 (Prototype) to evaluate the risk of bias in publications. Data from studies with a low risk of bias will be extracted and compiled into a narrative synthesis and summarized into tables and figures. If sufficient evidence is available, findings will be used to perform a meta-analysis.

**Supplementary Information:**

The online version contains supplementary material available at 10.1186/s13750-022-00274-y.

## Background

Aminoglycosides are potent bactericidal antibiotics that inhibit protein synthesis [1]. Aminoglycosides were first isolated from soil bacteria and some are frequently used in agricultural practices. Streptomycin, isolated from *Streptomyces griseus*, was the first aminoglycoside discovered in 1943. In addition to being the first isolated aminoglycoside, streptomycin is one of the three antibiotics currently registered by the EPA for use in plant agriculture in the United States. It is predominantly used in pear and apple orchards [2] and more recently in citrus groves [3]. Other aminoglycosides have been discovered over time including neomycin (1949, *S. fradiae*), kanamycin (1957, *S. kanamyceticus*), gentamicin (1963, *Micromonospora purpurea*), kasugamycin (1965, *S. kasugaensis*), tobramycin (1965, *S. tenebrarius*), apramycin (1967, *S. tenebrarius*), and sisomicin (1976, *Micromonospora inyoensis*). With the emergence and spread of streptomycin-resistant strains of the pathogen *Erwinia amylovora* in pear and apple orchards, there is increased adoption of kasugamycin as an alternative to streptomycin [4]. Aside from streptomycin and kasugamycin that are used in many countries around the world, gentamycin is also routinely used Mexico and Central America to control *Erwinia amylovora* in apple and pear as well as other diseases in vegetable crops caused by pathogenic *Pectobacterium*, *Pseudomonas*, *Ralstonia*, and *Xanthomonas* [2].

In addition, to direct application of antibiotics in plant agriculture, the use of manure for fertilization can serve as a route of antibiotic exposure in soil. Aminoglycosides are used to increase meat production by preventing infections or outbreaks in livestock [5]. The most commonly used aminoglycosides in veterinary medicine are neomycin, dihydrostreptomycin, apramycin, gentamicin, kanamycin, paromomycin and neomycin [6]. In general, aminoglycosides are poorly absorbed in the gastrointestinal tract of animals and when injected, they are rapidly excreted in urine without metabolic transformation [1]. Thus, manure from antibiotic-treated animals can be a significant source of aminoglycoside exposure in the soil environment. Manure would not only bring antibiotics, but also a different population of bacteria that have been selected by the treatment and often harbor antibiotic resistance genes [7]. Therefore, the impact of manure on soil bacteria might involve more complex dynamics than simple exposure to antibiotics. Other indirect routes of antibiotic exposure in agriculture include waste from humans excreted into water and soil through municipal wastewater, sewage sludge, and biosolid [8]. Among aminoglycosides, gentamicin, tobramycin and amikacin would be the antibiotics of concern as they are the most commonly used in humans [6, 9]. Despite the high likelihood of aminoglycoside exposure in agriculture, there is very little research on the relationship between aminoglycoside exposure and the soil microbiota. Likewise, the potential role of these antibiotics in the soil environment and in plant health is not well understood.

The microbial composition of the soil and roots plays a crucial role in maintaining the health of plants. Microorganisms are involved in organic matter turnover, nutrient release, stabilization of the soil structure, and soil fertility [1–3]. Moreover, microorganisms assist in nitrogen fixation [2] growth promotion [3], and stress tolerance in plants [4]. The microbiome associated with plant roots represents a distinct subset of the soil microbiome [1, 5–8]. This is largely attributed to the strong selective environment created by the host plant to assemble a particular guild of bacteria [9–11]. Those found in the direct vicinity of the roots represent the rhizospheric bacteria while those colonizing internal plant tissues represent the endosphere bacteria. Given the bactericidal nature of aminoglycosides, they can inhibit the growth of microorganisms and thus influence the composition of the soil and root microbiomes. Understanding aminoglycoside-induced alterations in the soil and plant microbiota could provide insight on the effect of aminoglycoside exposure and plant health.

In bacteria, aminoglycoside resistance is well documented and takes many different forms including enzymatic modification [12], target site modification via an enzyme or chromosomal mutation [13–15], and efflux [16–21]. Aminoglycoside resistance was also discovered in the model plant *Arabidopsis thaliana.* It is associated with an ATP binding cassette (ABC) transporter, WBC19 (ABCG19), capable of conferring resistance to kanamycin [22], neomycin, geneticin, and paromomycin [23]. Exposure to aminoglycosides therefore may provide a selective advantage to microorganisms harboring aminoglycoside resistance genes that can then become dominant in the microbial or plant community. More broadly, the existence and evolution of such resistance genes suggest that plant and soil bacteria exposure to aminoglycosides is prevalent.

Pathogen-suppressive soils have a microbial composition that promotes overall plant health. This might involve the creation of conditions that prevent the establishment of pathogens, allowing the establishment of pathogens but pathogens fail to cause disease, or allowing the establishment of pathogens that can cause disease but disease severity declines with the continued monoculture of the host crop [15]. Aminoglycoside exposure has the potential to impact soil suppressiveness. For example, *Pseudomonas* species were shown to play a major role in suppressing the pathogens *Penicillium digitatum and P. italicum* by producing the antifungal compound diacetylphloroglucinol (DAPG) [4, 24]. Clinical evidence also shows that *Pseudomonas* sp. are susceptible to aminoglycosides [25]. Aminoglycoside exposure, therefore, has the potential to impact plant microbial composition that could lead to reduction of soil suppressive bacteria.

To explore these questions, a scoping review was conducted by KB, BJ, and NC, after a consultation with a data librarian from the Atlanta University Center (AUC) Robert W. Woodruff Library. The results were promising enough for the investigators to remain committed to the idea. Using results from the scoping review, JC and MA drafted a first version of the review protocol with input from KB and BJ. Local conventional and organic farmers were invited for a focus group discussion to assess the relevance of the research question and strategies for identifying potential confounding factors and relevant literature. Six organic farmers joined the conversation, most likely because organic farmers take a more holistic approach to farming and wish to minimize unintended impacts of their management practices. During the focus group discussion, it was apparent that they were particularly interested in learning about the impact of antibiotics in soil and their potential role in disease suppression. The discussions resulted in the inclusion of compost as a potential confounding factor as it is frequently used for suppressing disease. The protocol was also modified to reflect suggestions made on relevant databases to include. In addition to other standard academic sources, the USDA (U.S. Department of Agriculture) and the EPA (Environmental Protection Agency) websites were included because they were recommended as excellent sources of information during our focus group. Once the authors agreed on the revisions to the protocol, it was submitted to *Environmental Evidence* for peer review.

During our discussion, it was also apparent that many organic farmers relied on results from single studies to guide their farming practices because they were unaware of a report that compiled all the relevant findings. A synthesis of the evidence on aminoglycoside exposure and the impact on the soil and plant root-associated microbiome via a systematic review can be a useful method for summarizing, evaluating, and reporting evidence from multiple studies to relevant stakeholders. The proposed systematic review will be conducted as part of an effort to assess the currently available literature for public stakeholders and inform future experiments for academic stakeholders to fill in the knowledge gap. We hope the review findings will have relevance to farmers interested in the effect of aminoglycoside exposure and plant health. While the primary stakeholders are farmers, the questions asked in this systematic review are also relevant for researchers interested in leveraging plant microbiomes for sustainable agriculture [26, 27]. There has been increased interest in plant microbiome research, with an emphasis on understanding plant associated microbes as a community, and in interaction with the host plant and the soil environment. However, an understanding of the role of antibiotics, and especially aminoglycosides in shaping these interactions is lacking. Such information is key for the development of strategies to increase plant health and decrease plant disease.

## Objective

The objective of the systematic review is to collate existing research on the impact of aminoglycoside exposure on the soil and plant root-associated microbiome. The review will also ascertain any knowledge gaps for future primary research areas.

### Primary question

What is the impact of aminoglycosides on the soil and plant root-associated microbiome composition?

### Secondary questions

Are aminoglycoside resistance genes enriched by aminoglycoside exposure?

Are soil pathogen suppressive bacteria reduced by aminoglycoside exposure?

### Components of the question

*Population:* Soil and plant root-associated microbiomes.

*Exposure:* aminoglycosides antibiotics.

*Comparator:* Control with no exposure (i.e., no aminoglycoside).

*Outcome:* Changes in overall species richness and diversity (microbial composition), changes of the resistome (i.e. the quantification of resistance genes), and the ability to suppress plant pathogens (e.g., Biomass reported as mg/kg; changes in banding patterns, or richness expressed as H' and S' indices, and abundance of resistance genes or suppressive pathogens reported as percentages).

## Methods

### Searching for articles

The systematic review will follow the Collaboration for Environmental Evidence (CEE) guidelines [26]. The search aims to retrieve a wide range of quantitative scientific evidence, i.e., research articles, conference presentations, or grey literature from specific websites covering the topic of aminoglycoside exposure and its impact on the soil and plant root-associated microbiome.

### Search terms and language

Some of the top consumers of aminoglycosides in agriculture are non-English speaking countries. To avoid eliminating articles that may include relevant information, we will not exclude articles based on language. We will use translation tools, assistance from the university librarian, and help from native academic speakers when available to translate any articles not written in English. Any articles that cannot be translated will be excluded from our analysis. To ensure we are assembling the most up-to-date information, the search will be updated as needed such that it will be less than 6 months old at the time of article submission.

The search terms/keywords that will be used to search for relevant literature are broken into two components: the population and the exposure and will be combined using Boolean operators “AND” and/or “OR”. To ensure a comprehensive search that yields at least 500 articles including benchmark articles, we will exclude the comparator and outcome from our search term combinations. Benchmark articles identified during the scoping are described in Table 1.

**Table 1:** List of benchmark articles

Author	Title	Reference
Davey et al. 1961	Translocation of streptomycin from Coleus leaves and its effect on rhizosphere bacteria	[28]
Ingham et al. 1984	Effects of streptomycin, cycloheximide, Fungizone, captan, carbofuran, cygon, and PCNB on soil microorganisms	[29]
McGhee et al. 2011	Evaluation of kasugamycin for fire blight management, effect on nontarget bacteria, and assessment of kasugamycin resistance potential in Erwinia amylovora	[30]
Badalucco et al. 1994	Activity and degradation of streptomycin and cycloheximide in soil	[31]
Duffy et al. 2014	Streptomycin use in apple orchards did not increase abundance of mobile resistance genes	[32]
Walsh et al. 2014	Restricted streptomycin use in apple orchards did not adversely alter the soil bacteria communities	[33]
Sanchez-Cid et al. 2021	Gentamicin adsorption onto soil particles prevents overall short-term effects on the soil microbiome and resistome	[34]
Sundin et al. 1995	Distribution of the streptomycin-resistance transposon Tn5393 among phylloplane and soil bacteria from managed agricultural habitats	[35]
Lee et al. 2005	Activity of some aminoglycoside antibiotics against true fungi, Phytophthora and Pythium species	[36]
Pan et al. 2019	Continuing impacts of selective inhibition on bacterial and fungal communities in an agricultural soil	[37]
Liu et al. 2021	Fate of bacterial community, antibiotic resistance genes and gentamicin residues in soil after three-year amendment using gentamicin fermentation waste	[38]
Blau et al. 2018	Soil texture-depending effects of doxycycline and streptomycin applied with manure on the bacterial community composition and resistome	[39]
Shade et al. 2013	Streptomycin application has no detectable effect on bacterial community structure in apple orchard soil	[40]
Zhang et al. 2017	Alterations in soil microbial communities caused by treatments with penicillin or neomycin	[41]
Lee et al. 2005	Activity of some aminoglycoside antibiotics against true fungi, Phytophthora and Pythium species	[36]

Population terms: soil, plant, root, endosphere, rhizosphere, microbiome, microorganism, bacteria.

Exposure terms: aminoglycoside, kanamycin, streptomycin, gentamicin, neomycin, tobramycin, kasugamycin, amikacin, dihydrostreptomycin, apramycin, paromomycin.

### Databases

The search will be conducted using these academic and non-academic databases:

1. Science Direct
2. Scopus
3. PubMed
4. Google Scholar (first five pages)
5. Web of Science (Core Collection database)

The final search string will be:

(“soil” OR “endosphere” OR “rhizosphere” OR “plant” OR “root”) AND (“microbiome” or “microorganism” or “bacteria”) AND (“aminoglycoside” OR “kanamycin” OR “gentamicin” OR “neomycin” OR “streptomycin” OR “tobramycin” OR “amikacin” OR “dihydrostreptomycin” OR “apramycin” OR “paromomycin”).

Specific antibiotic names will be excluded from the ScienceDirect search to adhere to boolean key term limits. Only articles in the press will be included from the Scopus search. Any articles still under review will be excluded from the Scopus search. Our search terms for the academic databases retrieved all 15 benchmark articles. Modifications may be necessary for websites. Any changes or deviations from the search strategy described in the protocol will be reported in our final full review report before submission.

### Specialist searches

The search will be conducted using the following organizational websites.

1. USDA (U.S. Department of Agriculture) PubAg
2. EPA (Environmental Protection Agency) National Service Center for Environmental Publications

The final search strategy for both websites will be:

(soil AND bacteria) AND (aminoglycoside OR kanamycin OR gentamicin OR neomycin OR streptomycin OR tobramycin OR amikacin OR dihydrostreptomycin OR apramycin OR paromomycin)

### Supplementary searches

JC will search for additional relevant articles by forward citation, a review of articles that cite relevant literature, and backward citation, articles cited by relevant literature, tracing on all articles included after the full-text screening. Relevant literature provided by stakeholders will also be included in our search.

## Article screening and study eligibility criteria

### Screening process

Search results from each academic and internet database will be exported to the Covidence systematic review management software. The management software will remove any duplicates present. No manual curation will be used to remove duplicates. Two reviewers will independently screen the title and abstracts of exported articles. Any disagreements or ambiguity will be identified through the Covidence software and resolved by review from a senior third party prior to full-text screening. Reviewers will independently complete a second level of screening to review the full text of the titles and abstracts meeting the eligibility criteria. Any disagreements or ambiguity will be identified through the Covidence software and resolved by review from a senior third party. Records of the number of articles excluded and reasons for their exclusion, at full-text level, will be provided as Additional file 1.

### Eligibility criteria

For a study to be included in the systematic review, it must meet the followingcriteria:

*Eligible population:* plant and root-associated microbiota: soil, endosphere, rhizosphere, or root.

*Eligible exposure:* aminoglycosides: kanamycin, gentamicin, neomycin, tobramycin, streptomycin, kasugamycin, amikacin, dihydrostreptomycin, apramycin, paromomycin.

*Eligible comparator:* no aminoglycoside.

*Eligible outcome:* Changes in overall species richness and diversity (microbial composition), change of the resistome (i.e. the quantification of resistance genes), and the ability to suppress plant pathogens (e.g., Biomass reported as mg/kg; changes in banding patterns, or richness expressed as H' and S' indices, and abundance of resistance genes or suppressive pathogens reported as percentages).

*Eligible study types:* Both field and laboratory control-interventions studies that include aminoglycoside exposure and show comparison to comparator i.e., no aminoglycoside application to soil.

*Eligible types of articles:* peer-reviewed research articles, conference presentations, other grey literature from the specified websites.

### Study validity assessment

Studies deemed eligible will be subjected to critical appraisal by two reviewers during the full-text screening. The CEE Critical Appraisal Tool Version 0.2 (Prototype) [19], modified to our review question, will be used for study validity. The CEE Critical Appraisal Tool will be used to assess and categorize each study's susceptibility to bias. Any disagreements will be resolved by a senior-level reviewer. For procedural independence, none of the reviewers will review any articles they have authored.

### Data coding and extraction strategy

Using Covidence, evidence tables of meta-data and data extraction (i.e., study findings) will be produced. For each screened study that fits the inclusion criteria and meets the study validity criteria, data will be extracted according to predetermined codes (Table 2). The following data will be coded for:

**Table 2:** Data coding

Code	Variable	Description
Bibliographic information	Author	Only last name of the first author and et. al, for the other colleagues
	Publication year	Year the paper was published
	Title	Full title of the paper
	Source of publication	Nature of the publication e.g., journal article, report, etc
Study location	Country	The country where the study was conducted
Study site	Field/laboratory	If the study is based in the field or laboratory
Soil type	Sandy/clay/silt/loamy	Taxonomic unit used in soil science to categorize soil characteristics
Plant species	Name	Taxonomic unit used to describe plants
Soil use	Agricultural/natural	Primary purpose of soil for crop use (agricultural) or no use (natural)
Experimental conditions	Light/dark	Light conditions for incubation period after exposure
	Temperature	Temperature in Celsius
	Duration	The length of time in days that soil was exposed to antibiotic before outcomes were measured
Properties of soil	pH	pH of soil
	Organic matter content	Proportion of organic matter of soil consisting of plants and animals in various stages of composition expressed as a percentage
	Moisture	Water content of soil expressed in terms of volume or weight
	Oxygen status	Oxygen present in the atmosphere in the soil expressed as concentrations
	Compost use	The use of compost to amend the soil
Antibiotic characteristics	Name	Name of aminoglycoside used for exposure
	Chemical formula	Formula describing chemical proportions of atoms making up aminoglycoside
	Concentration	Concentration of antibiotic expressed in terms of volume or weight
	Method of application	The way the microbiome was exposed to antibiotic (ex. manure, direct application, etc.)
	Limit of detection	Lowest concentration of antibiotic able to be detected in soil expressed in terms of volume or weight
	Duration of detection Frequency of application	Length of time in days that antibiotic is able to be detected in the soil The number of antibiotic exposures
Method for estimating microbial composition	PFLA/T-RFLP/DGGE	Experimental method used to determine the impact of aminoglycoside on microbial community
Reported levels of microbial composition	Reported levels of microbial composition	Biomass reported as mg/kg; changes in banding patterns, or richness expressed as H' and S' indices
Method for estimating resistance genes	Shotgun/high throughput	Meta-genomic sequencing method used to estimate resistance genes
Reported levels of resistance genes	Reported levels of resistance genes	Similarity or abundance reported as percentage
	Reported levels of resistance genes	Similarity or abundance reported as percentage
Method for estimating soil suppression	Shotgun/high throughput	Genomic sequencing method used to identify suppressive pathogens
Reported levels of suppressive pathogen	Reported levels of biological control agents	Similarity or abundance reported as percentage
Comparator	Control	Nature of the study/experiment control (i.e., no aminoglycoside)
Type of tillage	Depth of tillage used in agricultural practices	No-till, deep (deeper than 10 inches), medium-depth (5–10 inches), shallow (1–4 inches)

*Bibliographic information (author, year, title, source of publication).

*Study location (country).

*Study site (field/laboratory).

*Population (Soil, root, endosphere, rhizosphere).

*Soil type (i.e. sandy, loamy, clay, silt).

*Soil use (agricultural, natural).

*Type of tillage (no-till, deep (deeper than 10 inches), medium-depth (5–10 inches), shallow (1–4 inches)).

*Plant species (name).

*Experimental conditions (light vs dark, temperature, duration).

*Properties of soil (pH, organic matter content, moisture, oxygen status, compost use).

*Antibiotic characteristics (name, chemical formula, concentration, method of application, frequency of application, previous history of antibiotic application, and limits of detection).

*Method for evaluating microbial composition, resistance genes, and soil suppressiveness (e.g., Phospholipid Fatty Acid Analysis (PFLA), Terminal Restriction Fragment Length Polymorphism (T-RFLP), Denaturing Gradient Gel Electrophoresis (DGGE), Shotgun Sequencing, High throughput sequencing).

*Reported mean and standard deviation of microbial composition, resistance genes, and soil pathogen suppression for the control and intervention/experimental groups (e.g., Biomass reported as mg/kg; changes in banding patterns, or richness expressed as H' and S' indices, and abundance of resistance genes or suppressive pathogens reported as percentages).

*Comparator (description of the control with no exposure, i.e. no aminoglycosides).

Two reviewers will simultaneously and independently extract data from all studies deemed eligible based on the inclusion criteria. Any disagreements between the two reviewers will be resolved by a senior level third reviewer. If relevant data is either missing or ambiguous, the corresponding authors of those studies will be contacted. In the event of no response, we will indicate no response in our metadata and only report available data in our final analysis. If the amount of missing data is deemed to be substantial (> 50%), the study will be excluded from our final analysis.

### Potential effect modifiers and reasons for heterogeneity

The identified potential effect modifiers and sources for heterogeneity are listed below. Also mentioned are the methods of testing.

*Soil type (i.e. sandy, loamy, clay, silt) [subgroup analysis].

*Soil usage (agricultural, natural) [subgroup analysis].

* Type of tillage (no-till, deep (deeper than 10 inches), medium-depth (5–10 inches), shallow (1–4 inches)) [subgroup analysis].

*Plant species (name) [subgroup analysis].

*Experimental conditions (light vs dark), the method used [subgroup analysis], and temperature and duration [meta-regression].

*Properties of the soil (pH, organic matter content, moisture, oxygen content status, compost use) [meta-regression].

*Antibiotic characteristics (name), method of application, previous application of antibiotics [subgroup analysis] and concentration, half-life, sorption coefficient, frequency of application [meta-regression].

This list was developed based on a preliminary literature search performed by KB, BJ, and NC with input from JC. MA provided overall scientific expertise. If additional effect modifiers are identified during the review process, we will document any changes in a final list of effect modifiers.

### Data synthesis and presentation

After data extraction from all eligible studies, a narrative descriptive synthesis will be conducted on those demonstrating a low and medium risk of bias (based on study validity assessment), summarizing information in tables and figures. Summaries will be descriptive, outlining bibliographic information, study location, and site, population, soil characteristics, aminoglycoside characteristics, and experimental methods and conditions.

To assess the risk of publication bias, effects from individual studies will be visualized in funnel plots. Where evidence allows, efforts will be made to estimate the effect size of the outcome (i.e. microbial composition, antibiotic resistance genes, and soil suppression after aminoglycoside exposure relative to the comparator). We will attempt to estimate the standardized between-group mean difference (SMD_between_) and the standard error of SMD_between_ of each outcome previously described for every article deemed eligible by our inclusion criteria. We will pool the effect size using a frequentist random-effects statistical model and perform a meta-regression analysis to understand the relationship between aminoglycoside concentration and the SMD_between_. Meta-regressions or subgroup analyses of studies will be performed where studies report sources of heterogeneity. To test the robustness of reported findings, sensitivity analysis will be performed by including or excluding studies of unclear or high risk of bias. If data does not allow us to estimate a pooled effect size, we will report a narrative synthesis of the included articles and report the SMD_between_ point estimates for the individual studies.

In conclusion, the overall aim of this review is to identify, evaluate, and summarize all impacts of aminoglycoside exposure on soil and plant root-associated microbial composition, resistance genes, and soil suppressive species. The review will make the available evidence more accessible to researchers, farmers and environmental agencies invested in this information.

## Supplementary Information

**Additional file 1:** ROSES for Systematic Review Protocol Submission.

## Data Availability

All data generated or analyzed during this study are included in the published article and Additional files.
